# Development of a Novel Tetravalent Synthetic Peptide That Binds to Phosphatidic Acid

**DOI:** 10.1371/journal.pone.0131668

**Published:** 2015-07-06

**Authors:** Rina Ogawa, Kohjiro Nagao, Kentaro Taniuchi, Masaki Tsuchiya, Utako Kato, Yuji Hara, Takehiko Inaba, Toshihide Kobayashi, Yoshihiro Sasaki, Kazunari Akiyoshi, Miho Watanabe-Takahashi, Kiyotaka Nishikawa, Masato Umeda

**Affiliations:** 1 Department of Synthetic Chemistry and Biological Chemistry, Graduate School of Engineering, Kyoto University, Katsura, Kyoto, Japan; 2 Lipid Biology Laboratory, RIKEN, Wako, Saitama, Japan; 3 Department of Polymer Chemistry, Graduate School of Engineering, Kyoto University, Katsura, Kyoto, Japan; 4 Faculty of Life and Medical Sciences, Doshisha University, Kyotanabe, Kyoto, Japan; Nagoya University, JAPAN

## Abstract

We employed a multivalent peptide-library screening technique to identify a peptide motif that binds to phosphatidic acid (PA), but not to other phospholipids such as phosphatidylcholine (PC), phosphatidylethanolamine (PE), and phosphatidylserine (PS). A tetravalent peptide with the sequence motif of MARWHRHHH, designated as PAB-TP (phosphatidic acid-binding tetravalent peptide), was shown to bind as low as 1 mol% of PA in the bilayer membrane composed of PC and cholesterol. Kinetic analysis of the interaction between PAB-TP and the membranes containing 10 mol% of PA showed that PAB-TP associated with PA with a low dissociation constant of K_D_ = 38 ± 5 nM. Coexistence of cholesterol or PE with PA in the membrane enhanced the PAB-TP binding to PA by increasing the ionization of the phosphomonoester head group as well as by changing the microenvironment of PA molecules in the membrane. Amino acid replacement analysis demonstrated that the tryptophan residue at position 4 of PAB-TP was involved in the interaction with PA. Furthermore, a series of amino acid substitutions at positions 5 to 9 of PAB-TP revealed the involvement of consecutive histidine and arginine residues in recognition of the phosphomonoester head group of PA. Our results demonstrate that the recognition of PA by PAB-TP is achieved by a combination of hydrophobic, electrostatic and hydrogen-bond interactions, and that the tetravalent structure of PAB-TP contributes to the high affinity binding to PA in the membrane. The novel PA-binding tetravalent peptide PAB-TP will provide insight into the molecular mechanism underlying the recognition of PA by PA-binding proteins that are involved in various cellular events.

## Introduction

Phosphatidic acid (PA) is a central intermediate for the synthesis of glycerophospholipids, and PA produced by the enzymatic activity of phospholipase D plays crucial roles in a variety of cellular functions, such as vesicular trafficking, cytoskeletal organization, cell proliferation and signal transduction [1–4]. PA is shown to regulate the function of numerous proteins via direct binding to its specific binding sites as well as remodeling of the membrane structure by changing the physicochemical properties in a localized area [5, 6]. The chemical structure of PA consists of a diacylglycerol backbone and an anionic-phosphate head group attached as a phosphomonoester. The ionization constant of the phosphomonoester of PA displays two p*K*a values, a p*K*_a1_ of 3.2 and p*K*_a2_ of 7.9, in the bilayers composed of phosphatidylcholine (PC), while p*K*_a2_ shifts to 6.9 in the bilayers composed of PC and phosphatidylethanolamine (PE) [7]. This suggests that under physiological conditions, the ionization properties of PA are quite sensitive to the organization of lipid molecules in membranes, especially to coexisting lipids such as PE and cholesterol [7],[8]. Because of its unique ionization properties, PA is also suggested to act as a pH biosensor that coordinately regulates membrane biogenesis and cellular metabolisms by regulating the binding of the yeast transcription factor Opi1p to membranes in a pH-dependent manner [9].

More than 50 proteins have been shown to bind to PA in mammalian, plant and yeast cells [5, 10]. Although the PA-binding domains of protein kinases, protein phosphatases, and transcription factors have been determined, no consensus PA-binding motif was identified among these proteins, in striking contrast to other lipid-binding domains such as C2, PH, FYVE and PX that are conserved among various acidic phospholipid-binding proteins [11]. The PA-binding domains of Opi1p, mammalian Raf-1 kinase, the Rac1 exchange factor DOCK2, and t-SNARE Spo20p have been well studied: a cluster of basic amino acids have been shown to play crucial roles in the interaction with PA. These consist of two regions, KRQK and KKR, at residues 109–112 and 136–138, respectively, of Opi1p [12]; RKTR at residues 398–401 of Raf-1 [13]; three regions, VREM, RPR, and KKR, at residues 1618–1621, 1631–1633 and 1697–1699, respectively, of DOCK2 [14]; and two regions, KLK and RNK, at residues 66–68 and 71–73, respectively, of Spo20p [15]. The PA-binding domains of Raf-1, Spo20P and DOCK2 have also been employed for the generation of biosensors that visualize the distribution of PA in mammalian, yeast and plant cells [16, 17]. A recent study of yeast Yas3p, an Opi1 family transcription repressor, showed that Yap3P has two regions that bind to PA and phosphoinositides, and the binding of Yap3p to PA-containing vesicles is significantly enhanced by the presence of PE, suggesting that the cooperative effects among lipid molecules in biological membranes also affect the PA-protein interactions [18]. Although the positively charged amino-acid and adjacent hydrophobic amino-acid residues of the PA-binding proteins are predicted to form an amphipathic helix that may be crucial for PA binding, the exact nature of the lipid-protein interaction remains to be elucidated [19].

We have established various phospholipid-binding probes, including monoclonal antibodies against each of the following: phosphatidylinositol 4,5-bisphosphate (PI(4,5)P_2_) [20], phosphatidylserine (PS) [21], phosphatidylcholine (PC) [22]; a tetracyclic peptide of 19 amino acids (Ro09-0198) that binds specifically to phosphatidylethanolamine (PE) [23]; and the earthworm protein lysenin that binds specifically to sphingomyelin [24]. These probes have provided useful tools to explore the localization, metabolism, and molecular function of phospholipids [25–28]. In the present study, we applied the multivalent peptide-library screening technique to identify a peptide motif that interacts with PA in bilayer membranes. This technique was first utilized to identify the tetravalent peptide that bound specifically to the globotriaosyl ceramide (Gb3)-binding sites of Shiga toxin (Stx2) [29]. The library of tetravalent peptides carrying identical MAXXXXXXX sequence, where X represents a random amino acid residue, was selected for the binding to the Stx2 B-subunit (2BH) but not to the mutated form of 2BH (2BH-W33A) lacking the ability to recognize Gb3. Among the four candidate peptides, the tetravalent peptide with an MAPPPRRRR sequence, designated as PPP-tet, bound to the Gb3-binding sites of Stx2 with extremely high affinity and inhibited the Stx2-mediated bloody diarrhea and hemorrhage colitis [30]. The high affinity binding was shown to be partly due to the multivalent interactions between the PPP-tet and the pentameric B-subunit of Stx2 [29]. The estimated surface area (~6.3 nm^2^) covered by the 7 amino acid span of the tetravalent peptide roughly corresponds to the surface area occupied by 8 ~ 10 molecules of phospholipids in fluid-state membranes [31, 32]. This estimation prompted us to apply the tetravalent peptide-library screening technique to develop a novel probe that may specifically recognize the molecular structure or a particular surface distribution of PA molecules in bilayer membranes.

Here we have developed a tetravalent peptide with the sequence motif of MARWHRHHH, designated as PAB-TP (phosphatidic acid-binding tetravalent peptide), which can bind to as low as 1 mol% of PA in bilayer membranes. A series of analyses revealed the amino acid residues responsible for the interaction and the molecular mechanisms underlying the high affinity peptide-PA interactions in bilayer membranes.

## Materials and Methods

### Materials

1,2-dioleoyl-*sn*-glycero-3-phosphate (DOPA), 1,2-dioleoyl-*sn*-glycero-3-phospho- L-serine (DOPS), 1,2-dioleoyl-*sn*-glycero-3-phospho-(1'-*rac*-glycerol) (DOPG), 1,2-dioleoyl-*sn*-glycero-3-phospho-(1'-myo-inositol) (DOPI), 1,2-dioleoyl-*sn*-glycero- 3-phospho-(1'-*myo*-inositol-4'-phosphate) (PI(4)P), 1,2-dioleoyl-*sn*-glycero-3-phospho- (1'-*myo*-inositol-5'-phosphate) (PI(5)P), 1,2-dioleoyl-*sn*-glycero-3-phospho- (1'-*myo*-inositol-4',5'-bisphosphate) (PI(4,5)P_2_), 1,2-dioleoyl-*sn*-glycero-3- phosphocholine (DOPC), 1,2-dimyristoyl-*sn*-glycero-3-phosphocholine (DMPC), L-α-phosphatidylcholine (Egg, Chicken) (EggPC), 1,2-dioleoyl-*sn*-glycero-3- phosphoethanolamine (DOPE), N-oleoyl-ceramide-1-phosphate (C1P), 1,2-dioleoyl-*sn*-glycero-3-phosphoethanolamine-N- (biotinyl) (biotin-DOPE), and 1,2-dipalmitoyl-*sn*-glycero-3-phosphoethanolamine-N- (cap biotinyl) (biotin-DPPE) were purchased from Avanti Polar Lipids (Alabaster, AL). Cholesterol and GM3 were obtained from Wako Pure Chemical Industries (Osaka, Japan) and Matreya LLC (Pleasant Gap, PA), respectively. Tetravalent peptides, monomer peptides, and the tetravalent peptide library were synthesized using N-α-FMOC-protected amino acid and standard BOP/HOB coupling chemistry as described previously [29]. The four peptide-chains present in a given tetravalent peptide were elongated at the same time from the amino groups of the core polylysine (Fmoc MAP resin; Applied Biosystems, Foster City, CA).

### Preparation of large unilamellar vesicle (LUV)

A chloroform solution of lipid mixtures was dried at the bottom of a flask for 15 min and evaporated in vacuo overnight. The dried lipid film was hydrated with Tris-buffered saline (TBS; 10 mM Tris-HCl, pH 7.0, 150 mM NaCl). The resulting multilamellar vesicles were put through five freeze/thaw cycles and then extruded 20 times through a polycarbonate filter with a pore diameter of 100 nm using an Avanti Mini-Extruder.

### Peptide library screening

**Fig 1:**
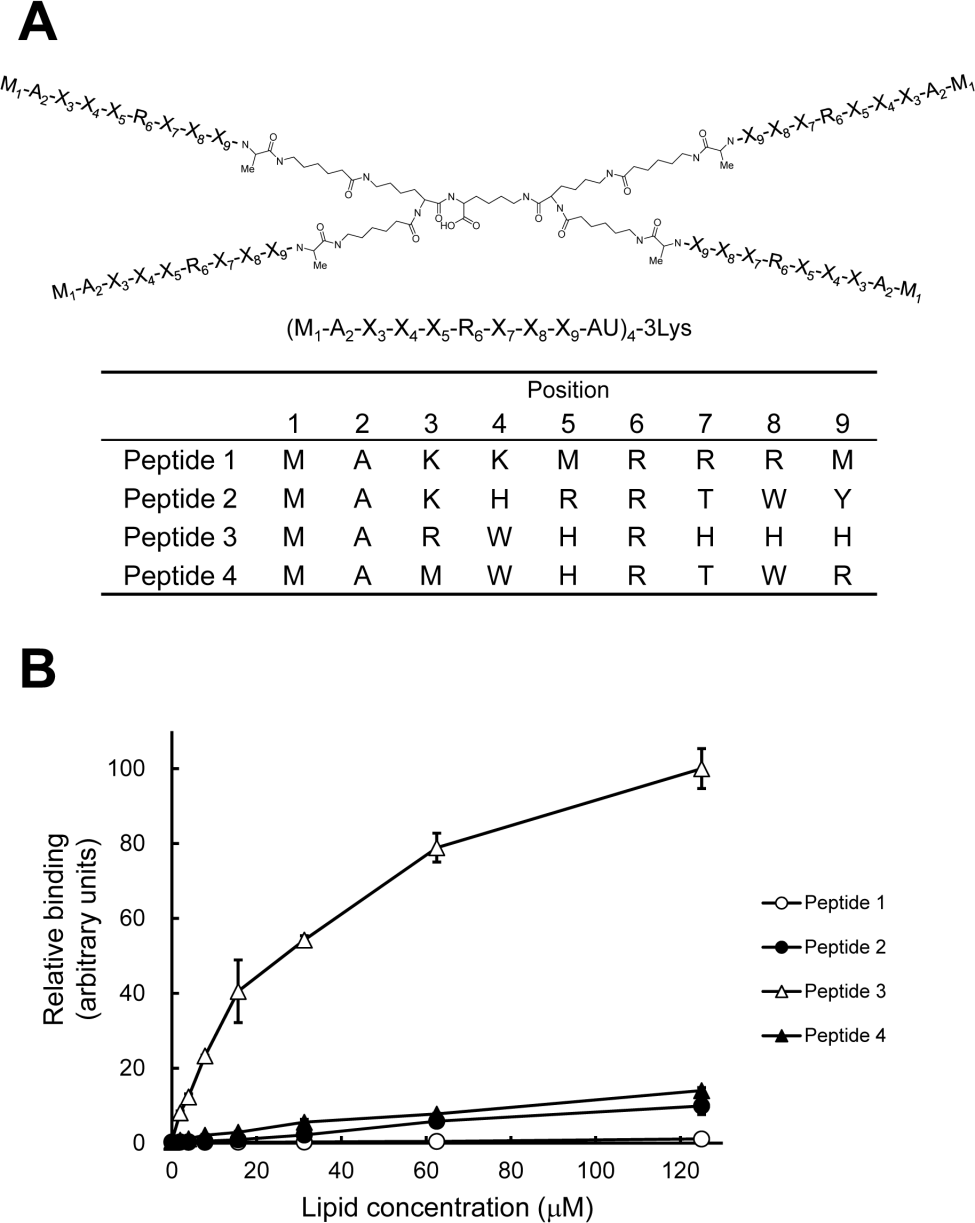
Identification of PA-binding peptide motif using tetravalent peptide libraries. (A) The tetravalent peptide library was comprised of compounds with 4 randomized peptides of sequence M_1_-A_2_-X_3_-X_4_-X_5_-R_6_-X_7_-X_8_-X_9_-AU (U; amino hexanoic acid), where X at positions 3–5 and 7–9 indicates any amino acids except cysteine. Screening of the library was performed to identify compounds that bound to vesicles containing PA but not to vesicles without PA. Sequences of PA binding peptides (peptide 1 to 4) were determined by comparing relative abundance of amino acids in each degenerate position of PA-, PC-, and PS-bound peptides. (B) Binding of the tetravalent peptides to LUVs composed of DOPA/DOPC/biotin-DOPE/cholesterol (10:58:2:30) was examined by the SPVB assay (mean ± SE, n = 3). The binding of LUVs at the concentration of 125 μM to peptide 3 was represented as 100 (arbitrary units).

Screening of the tetravalent peptide libraries was performed as described previously [29]. In brief, the four peptide-chains present in a given tetravalent peptide (Fig 1A) were elongated at the same time from the amino groups of the core polylysine. The Met-Ala sequence at the amino terminus of the library peptides was included to verify that peptides from this mixture were being sequenced and to qualify the peptides, and the Arg residue at position 6 was fixed in the library peptides. The predicted degeneracy of a randomize peptide with 6 degenerate positions is 19^6^ (47 million). LUVs composed of either DOPA/DMPC/biotin-DOPE/cholesterol (10:38:2:50), DMPC/biotin-DOPE/cholesterol (48:2:50) or DOPS/DMPC/biotin-DOPE/cholesterol (10:38:2:50) were incubated with Streptavidin-Agarose beads from Sigma Aldrich (St. Louis, MO) and washed with PBS. Vesicles containing DMPC were used for screening to avoid disruption of the membrane integrity during incubation with the library peptides and washing procedures. The LUVs bound to the beads were incubated with the library peptides for 16 h at 4°C. After washing with PBS, peptides bound to the LUVs were separated by SDS-PAGE and sequenced on an Applied Biosystems model 477A protein sequencer. To calculate the relative amino acid preference at each degenerate position, the corrected quantities of amino acids were compared with those obtained using LUVs of different phospholipid compositions. The relative abundance of individual amino acids at the degenerate positions reflects the relative abundance of high-affinity peptides containing these amino acids [29].

### Solid phase vesicle-binding (SPVB) assay

96-well microtiter plates (Greiner Bio-One GmbH, Kremsmünster, Austria) were coated with 50 μL of PAB-TP (10 μg/mL) in TBS. After blocking with TBS containing 3% bovine serum albumin (BSA), PAB-TP coated on the solid phase was incubated with various concentrations of LUVs containing 2 mol% of biotin-DOPE (details of composition are described in figure legends) in TBS containing 1% BSA for 1 h at room temperature. After washing with TBS, the wells were incubated with HRP-conjugated streptavidin diluted 1/1000 with TBS containing 1% BSA for 45 min. The intensity of the color developed with *o*-phenylenediamine as the substrate for 3 to 20 min was measured using an Infinite F200 PRO microplate reader (TECAN). Appropriate reaction time for the color development, which was constant in individual curves of each Figure, was employed to avoid the saturation of peroxidase reaction.

### Fluorescence measurement

Fluorescence measurements were carried out using an LS55 Fluorescence Spectrometer (Perkin Elmer). 10 μg/ml (1.6 μM) of PAB-TP was incubated with LUVs (0–160 μM) in TBS for 1 h at 25°C. Tryptophan emission fluorescence was recorded at 25°C from 300 to 420 nm using a 280 nm excitation wavelength [33].

### Quartz crystal microbalance with dissipation (QCM-D) measurement

QCM-D measurement was performed by using a Q-Sense E1 (Q-sense, Göteborg, Sweden) as described previously [34]. The sensor coated with SiO_2_ (QSX303; Q-sense) was cleaned in 2% SDS solution (30 min at 50°C), rinsed with Milli-Q water, dried under a N_2_ stream and treated with UV-ozone cleaner (PC450; Meiwafosis, Japan). The sensor was oscillated at the resonance frequency of 5 MHz and at 6 overtone harmonics (15, 25, 35, 45, 55 and 65 MHz). The difference of frequency (Δf) and dissipation (ΔD) were recorded. The 25, 35, 45 and 55 MHz harmonics were used for the analysis.

The sensor was equilibrated with TBS before measurement. The solution was subsequently flowed onto the sensor surface. A supported lipid bilayer (SLB) was formed on the sensor surface by flowing small unilamellar vesicles (SUVs) (0.2 mM total lipids) composed of EggPC/biotin-DPPE (99:1). After the surface was rinsed with TBS to remove excess SUVs, 2 μM streptavidin was introduced to be fixed on the lipid membrane. The streptavidin layer was rinsed with TBS and then LUVs (0.2 mM total lipids) composed of DOPA/DOPC/biotin-DOPE/cholesterol (10:58:2:30) or DOPC/biotin-DOPE/cholesterol (68:2:30) were introduced. To determine the interaction between PAB-TP and LUVs, increasing concentrations of PAB-TP (7.8–1000 nM) were introduced onto the LUV layer to induce the binding. Between the flows of PAB-TP, TBS was introduced to induce the release of PAB-TP. *K*_D_, *k*_off_, and *k*_on_ were calculated from the profile of PAB-TP binding and release.

### Vesicle size and zeta potential measurement

The particle sizes and zeta potentials of vesicles were determined at 25°C using a Zetasizer Nano ZS (Malvern Instruments Ltd., UK) [35]. The concentration of the sample was kept constant at 1.0 mM.

## Results and Discussion

### Identification of a tetravalent peptide that binds to PA

**Fig 2:**
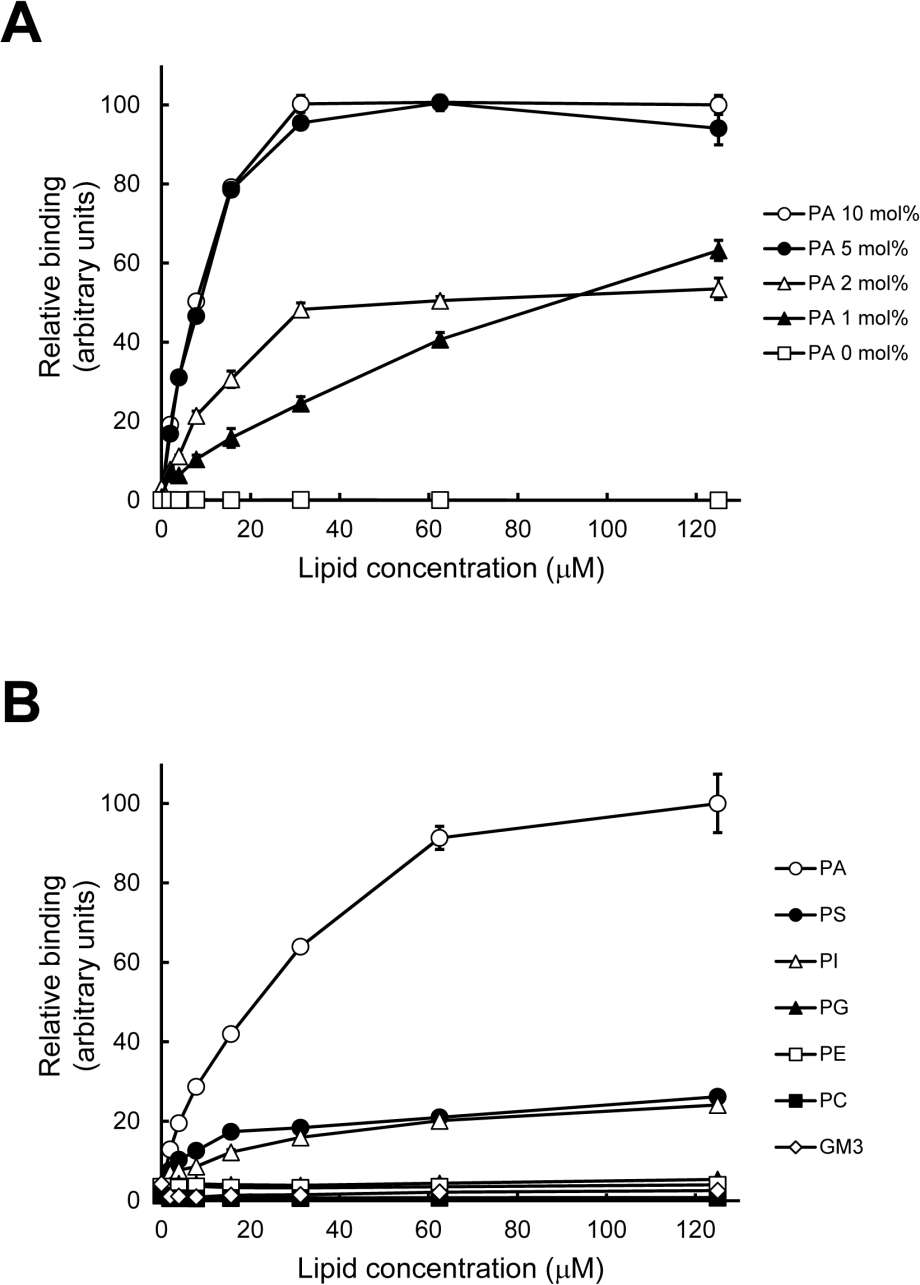
Analysis of binding of PAB-TP to phospholipids by the solid phase vesicle-binding assay. (A) Binding of PAB-TP to LUVs composed of DOPA/DOPC/biotin-DOPE/cholesterol (10:58:2:30), (5:63:2:30), (2:66:2:30), (1:67:2:30), and (0:68:2:30) was examined by the SPVB assay (mean ± SE, n = 3). (B) Binding of PAB-TP to LUVs composed of DOPC, biotin-DOPE, cholesterol, and indicated lipid (58:2:30:10) was examined by the SPVB assay (mean ±SE, n = 3). (A, B) The binding of LUVs composed of DOPA/DOPC/biotin-DOPE/cholesterol (10:58:2:30) at the concentration of 125 μM to PAB-TP was represented as 100 (arbitrary units).

In this study, we employed a peptide library composed of tetravalent peptides containing a polylysine core bifurcating at both ends with six randomized residues and fixed methionine, alanine, and arginine at positions, 1, 2, and 6, respectively [29] (Fig 1A). The peptide library was screened for its ability to bind to vesicles containing PA but not to vesicles without PA. As previously reported for screening of the Stx2-binding peptide [29], we determined the relative amino acid preference at each position, and identified four candidate sequence motifs: MAKKMRRRM, MAKHRRTWY, MARWHRHHH, and MAMWHRTWR (Fig 1A). The tetravalent forms of these peptides with the same core structure were synthesized and were evaluated for their ability to bind to vesicles containing PA by the solid phase vesicle-binding (SPVB) assay. In this assay, the peptides coated on the solid phase were incubated with LUVs composed of DOPA, DOPC, biotin-DOPE, and cholesterol at the molar ratio of 10:58:2:30, followed by quantification of the binding of vesicle to the peptide using HRP-streptavidin. As shown in Fig 1B, the tetravalent peptide 3 with the sequence motif of MARWHRHHH bound effectively to the PA-containing vesicle, while only weak or no significant bindings were observed with other peptides. We designated the tetravalent peptide 3 with the sequence of MARWHRHHH as PAB-TP (phosphatidic acid-binding tetravalent peptide) and employed it for further analyses. In the SPVB assay, PAB-TP did not bind to the vesicles without PA, but bound effectively to the vesicles containing as low as 1 mol% of PA (Fig 2A). PAB-TP did not bind to the vesicles containing PE, PG, or ganglioside GM3, but exhibited a weak cross-reactivity with vesicles containing PS or PI (Fig 2B).

Our attempts to perform a kinetic analysis of the interaction between PAB-TP and PA-containing membranes using surface plasmon resonance measurement were hampered by non-specific adhesion of the peptide to sensor chips. To overcome this problem, we employed the quartz crystal microbalance (QCM) method, and coated the sensor chips with multiple membrane layers to suppress the non-specific adhesion of the peptide. The kinetic analyses showed that PAB-TP was associated with a membrane surface containing 10 mol% of PA with a *k*_on_ value of 5.2 ± 0.1 x 10^3^ M^-1^ s^-1^ and *k*_off_ value of 2.0 ± 0.3 x 10^−4^ s^-1^, giving a very low dissociation constant of *K*_D_ = 38 ± 5 nM (S1 Fig). The kinetic parameters for the binding of PAB-TP to membranes composed of PC and cholesterol were as follows: *k*_on_ 5.5 ± 0.0 x 10^3^ M^-1^ s^-1^, *k*_off_ 3.2 ± 0.2 x 10^−3^ s^-1^, and *K*_D_ 0.58 ± 0.03 μM.

### Effect of PE and cholesterol on the binding of PAB-TP to PA-containing vesicles

**Fig 3:**
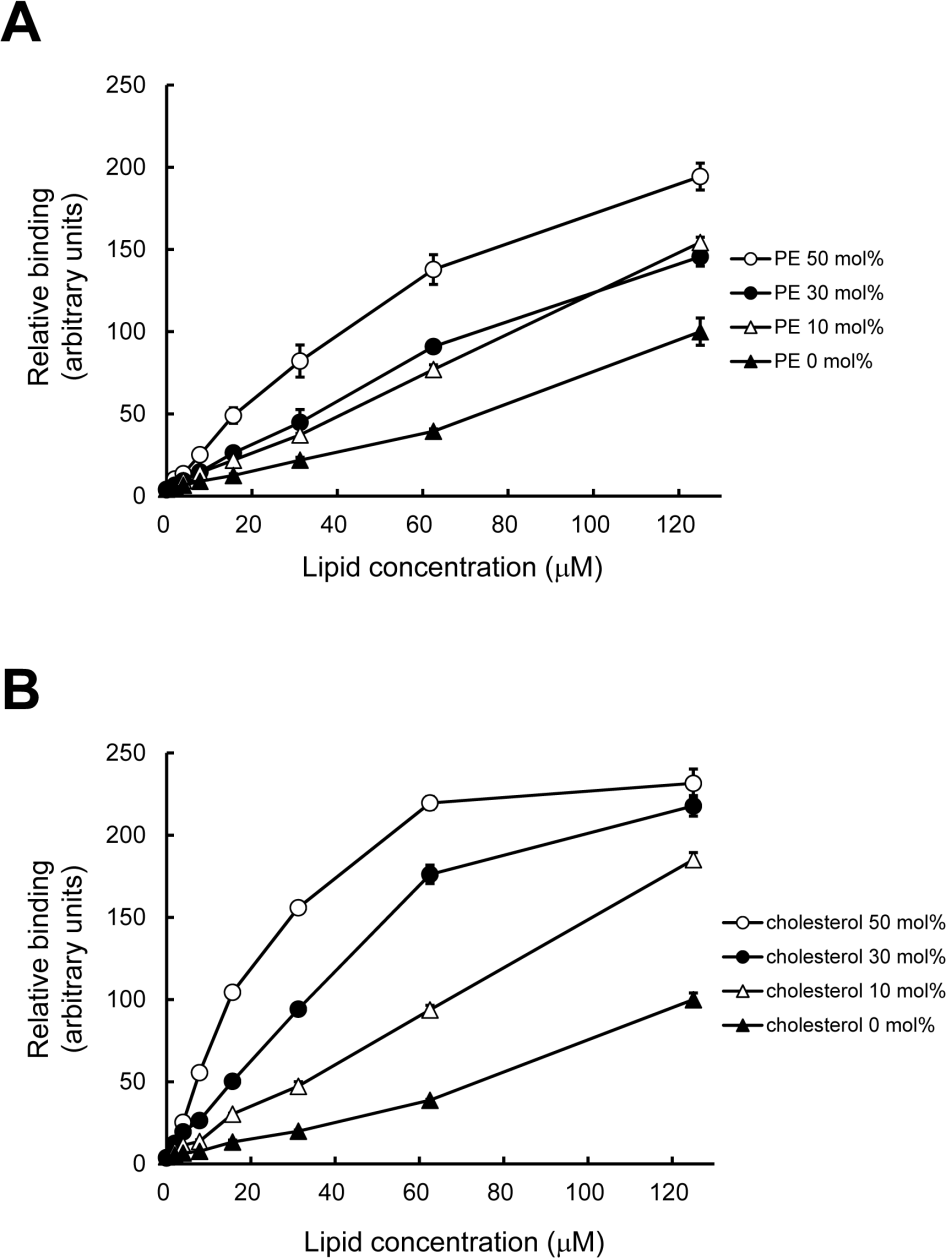
Effect of PE and cholesterol on the binding of PAB-TP to PA-containing vesicles. (A) Binding of PAB-TP to LUVs composed of DOPA/DOPC/biotin-DOPE/DOPE (10:88:2:0), (10:78:2:10), (10:58:2:30), and (10:38:2:50) was examined by the SPVB assay (mean ± SE, n = 3). (B) Binding of PAB-TP to LUVs composed of DOPA/DOPC/biotin-DOPE/cholesterol (10:88:2:0), (10:78:2:10), (10:58:2:30), and (10:38:2:50) was examined by the SPVB assay (mean ± SE, n = 3). (A, B) The binding of LUVs composed of DOPA/DOPC/biotin-DOPE (10:88:2) at the concentration of 125 μM to PAB-TP was represented as 100 (arbitrary units).

Since Kooijman et al. have shown that the ionization of PA is enhanced by coexistence of PE and cholesterol [7], we next analyzed the effect of PE and cholesterol on the binding of PAB-TP to PA. As shown in Fig 3A, the PAB-TP binding to PA in the PC membranes was significantly enhanced by the presence of PE. The coexistence of cholesterol also drastically increased the binding of PAB-TP to the PA-containing vesicles (Fig 3B). To examine the effect of PE and cholesterol on the electrostatic properties of the membranes, we measured the zeta potential of the PA-containing vesicles with various amounts of PE and cholesterol. A significant increase in the negative zeta potential was observed with the presence of PE in the PA-containing vesicles (Table 1), reflecting the formation of a hydrogen bond between PE and phosphomonoester of PA that causes further deprotonation and ionization of PA [7]. The negative zeta potential of the PA-containing vesicles was also remarkably increased in a dose-dependent manner by cholesterol (Table 1). Addition of cholesterol into the vesicles in the absence of PA did not significantly affect the zeta potential (-0.4±0.4 and -1.7±0.4 mV for vesicles composed of only DOPC and DOPC/cholesterol (50:50), respectively), indicating that the effect of cholesterol reflects the increased ionization of PA. Furthermore, the size of the PA-containing vesicle was not significantly affected by the presence of PE or cholesterol (Table 1). These results suggest that the presence of PE or cholesterol increases the negative surface charge of the PA-containing vesicles, causing the enhanced binding of PAB-TP to the membrane. Although PE and cholesterol caused the comparable increase in the negative zeta potential, cholesterol has a more striking effect on the binding of PAB-TP to the PA-containing vesicles. As proposed by Kooijman et al. it is likely that cholesterol has additional effects such as induction of negative curvature stress in the planar lipid bilayers that enhance the insertion of hydrophobic residues into the hydrophobic interior of lipid bilayer [7]. Taken together, these results suggest that PAB-TP can distinguish the cholesterol and PE-mediated changes in the ionization properties of PA molecules as well as the microenvironment of PA molecules such as curvature stress of the lipid bilayer.

**Table 1:** Zeta potential and size of LUVs.

Lipid composition (mol%)					
DOPA	DOPC	cholesterol	DOPE	Zeta potential (mV)	Vesicle size (nm)
10	90	0	0	-5.6 ± 0.8	111.1 ± 0.5
10	80	10	0	-9.7 ± 0.4	166.6 ± 1.6
10	60	30	0	-10.9 ± 0.7	147.7 ± 8.2
10	40	50	0	-16.3 ± 0.0	130.8 ± 1.7
10	80	0	10	-8.7 ± 0.8	124.9 ± 0.8
10	60	0	30	-8.0 ± 1.0	126.2 ± 0.8
10	40	0	50	-11.5 ± 0.3	126.0 ± 0.6

Zeta potential (mV) and vesicle size of LUVs composed of indicated lipids were determined using a Zetasizer Nano ZS (mean ± SE, n = 3).

### Inhibition analysis of SPVB assay

**Fig 4:**
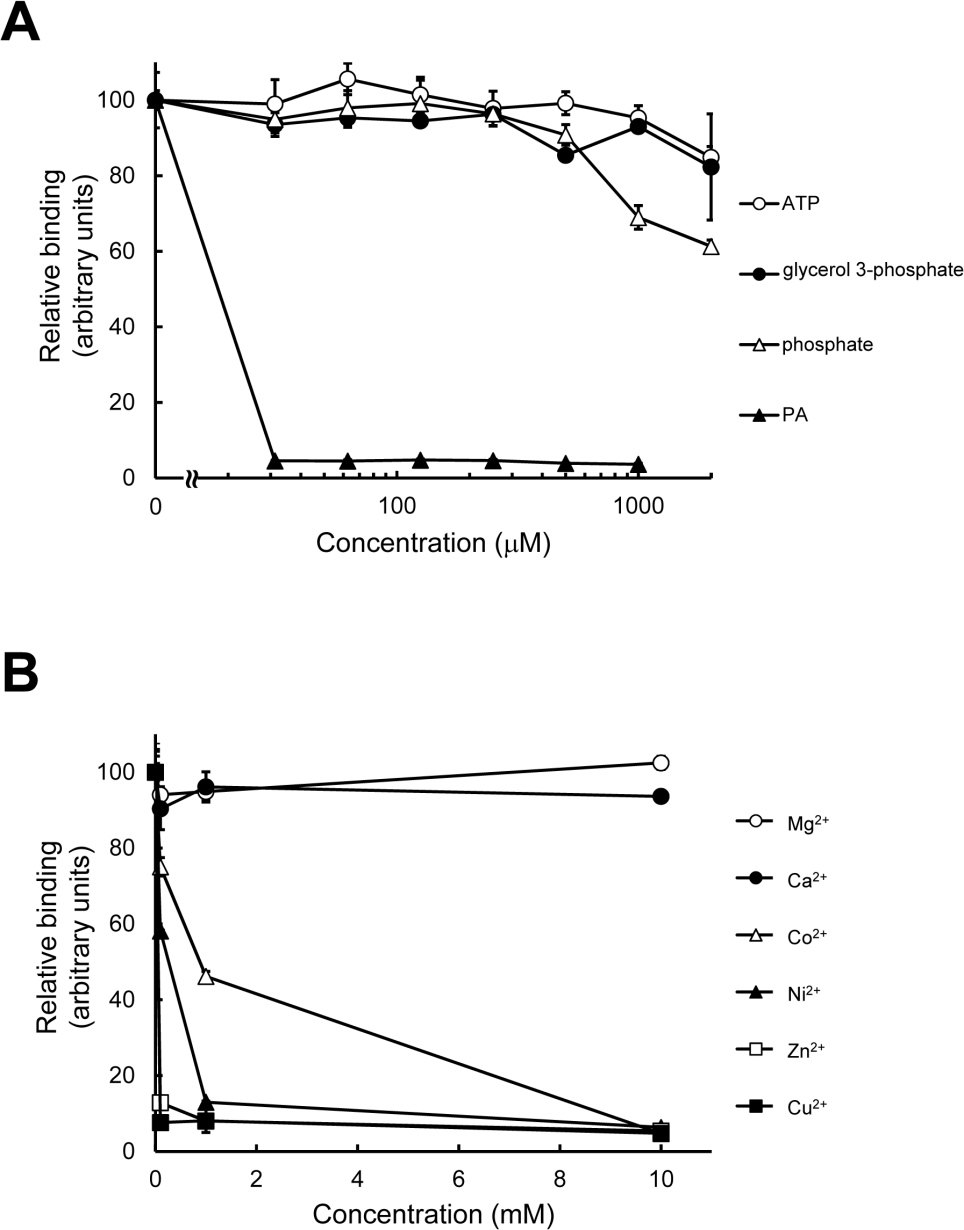
Inhibitory effect of soluble head groups and divalent cations on binding of PAB-TP to vesicles containing PA. (A) Binding of PAB-TP to LUVs (31.25 μM total lipids) composed of DOPA/DOPC/biotin-DOPE/cholesterol (10:58:2:30) in the presence of indicated concentrations of ATP, glycerol 3-phosphate, phosphate, and LUVs composed of DOPA was examined by the SPVB assay. (mean ± SE, n = 3). (B) PAB-TP coated on the solid phase was incubated with MgCl_2_, CaCl_2_, CoCl_2_, NiCl_2_, ZnCl_2_, and CuCl_2_ (0.1, 1, 10 mM). After removal of unbound divalent cations, binding of PAB-TP to LUVs (31.25 μM total lipids) composed of DOPA/DOPC/biotin-DOPE/cholesterol (10:58:2:30) was examined by the SPVB assay. (mean ± SE, n = 3). The binding of LUVs to PAB-TP in the absence of competitor (A) or divalent cation (B) was represented as 100 (arbitrary units).

The interaction between PAB-TP with the water-soluble head group of PA and other soluble compounds carrying the phosphomonoester group was examined by inhibition analysis of the SPVB assay. LUV composed of PA effectively inhibited the binding of PA-containing vesicles to PAB-TP, but no significant inhibition was observed with soluble compounds such as glycerol 3-phosphate and ATP (Fig 4A). This result suggests that a secondary interaction between PAB-TP and the hydrophobic fatty acyl chains of the PA molecules is required for the stable complex formation. The presence of divalent cations such as Cu^2+^, Zn^2+^, Ni^2+^, and Co^2+^ that associate with histidine also effectively inhibited the binding, while neither Ca^2+^ nor Mg^2+^ had a significant effect on the binding, suggesting that the histidine residues of PAB-TP may play a critical role in the binding to PA (Fig 4B).

### Amino acid residues involved in the PA-binding

**Fig 5:**
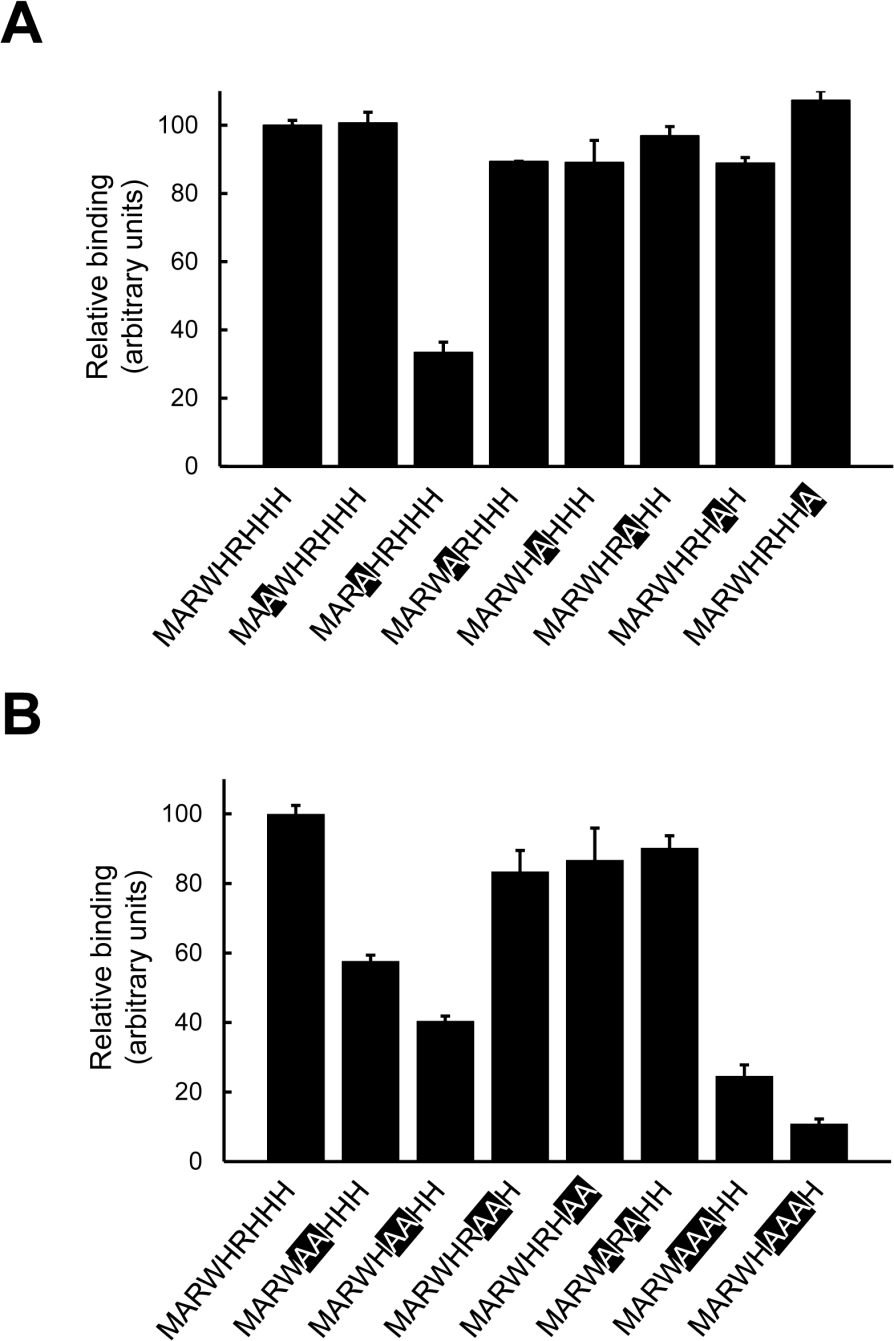
Identification of amino acid residues involved in the PA-binding. (A, B) Binding of alanine mutants of PAB-TP to LUVs (125 μM total lipids) composed of DOPA/DOPC/biotin-DOPE/cholesterol (10:58:2:30) was examined by the SPVB assay. Substituted amino acids were indicated by outline characters. (mean ± SE, n = 3). The binding of LUVs to PAB-TP (MARWHRHHH) was represented as 100 (arbitrary units).

To evaluate the possible involvement of each amino acid residue in the binding, the effect of alanine replacement of PAB-TP on its binding to PA-containing vesicles was examined. Alanine replacement of the Trp residue at position 4 significantly reduced the binding, while no significant change in the binding was observed with the replacement of other residues (Fig 5A). Since the binding was not significantly affected by the alanine replacements of a single His or Arg residue, we next examined the binding of double replacement mutants to PA-containing vesicles. As shown in Fig 5B, double replacement of the consecutive His and Arg residues (H5/R6 and R6/H7) significantly diminished the binding, while those of His residues (H5/H7) had no significant effect. Triple replacement of either H5/R6/H7 or R6/H7/H8 also strongly affected the binding. The wavelength of maximum fluorescence of triple replacement mutants (excitation 280 nm) in the absence of lipids is comparable to that of PAB-TP, indicating that abolishment of binding properties in triple replacement mutants was not due to the self-segregation of the peptide chains (data not shown) (see below). These results suggest that the Trp residue and the couplings of consecutive Arg and His residues play a critical role in the association of TAB-TP to the PA molecules in the membrane.

### Fluorescence analysis of phospholipid specificity

**Fig 6:**
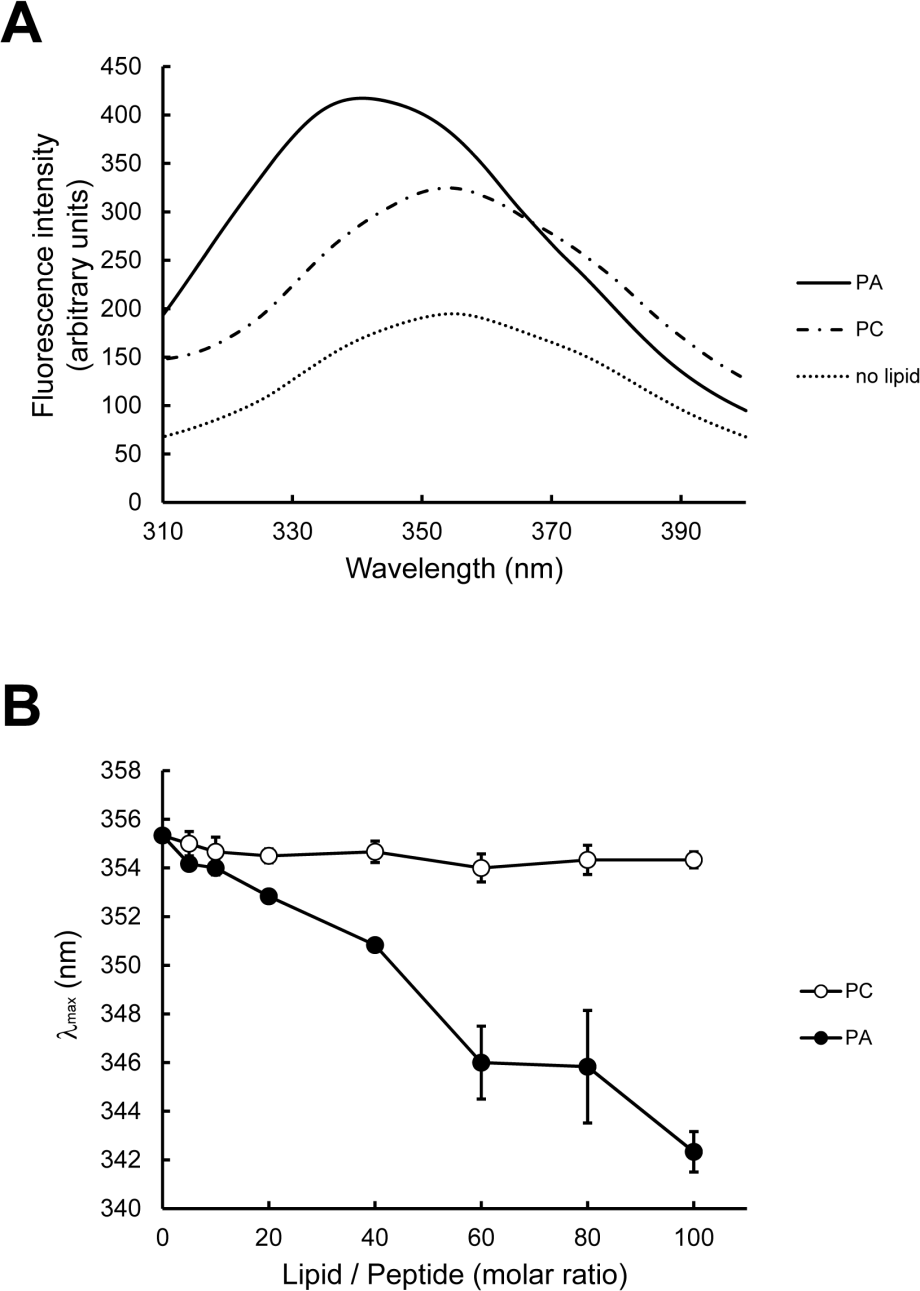
Fluorescence measurement of tryptophan residues. (A) PAB-TP was incubated with or without LUVs (160 μM total lipids) composed of DOPA/DOPC/cholesterol (10:60:30) and DOPC/cholesterol (70:30) for 1h at 25°C. Fluorescence spectra were obtained with the excitation wavelength at 280 nm. (B) PAB-TP was incubated with LUVs (0–160 μM total lipids) composed of DOPA/DOPC/cholesterol (10:60:30) and DOPC/cholesterol (70:30) for 1h at 25°C. Fluorescence spectra were obtained with the excitation wavelength at 280 nm and the wavelength of maximum fluorescence (λ_max_) was plotted against the molar ratio of lipid to peptide. (mean ± SE, n = 3).

**Fig 7:**
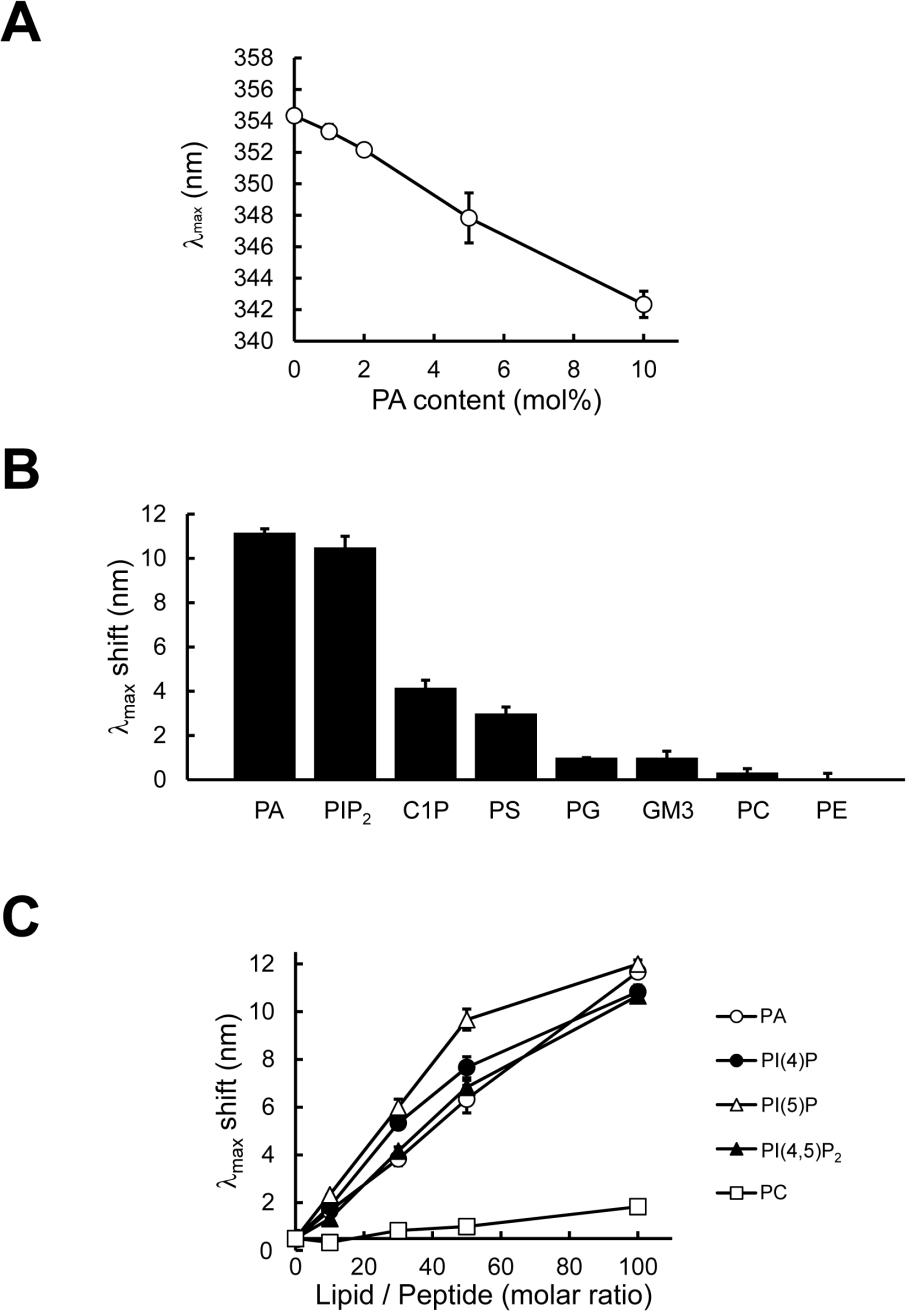
Analysis of PAB-TP by fluorescence measurement of tryptophan residues. (A) PAB-TP was incubated with LUVs (160 μM total lipids) composed of DOPA/DOPC/cholesterol (10:60:30), (5:65:30), (2:68:30), (1:69:30), and (0:70:30) for 1h at 25°C. Fluorescence spectra were obtained with the excitation wavelength at 280 nm and the wavelength of maximum fluorescence (λ_max_) was plotted against PA content (mol%). (mean ± SE, n = 3). (B) PAB-TP was incubated with LUVs (160 μM total lipids) composed of DOPC, cholesterol, and indicated lipids (60:30:10) for 1h at 25°C. Fluorescence spectra were obtained with the excitation wavelength at 280 nm and blue shift in the wavelength of maximum fluorescence (λ_max_) was calculated. (mean ± SE, n = 3). (C) PAB-TP was incubated with LUVs (0–160 μM total lipids) composed of DOPC, cholesterol, and indicated phospholipid (60:30:10) for 1h at 25°C. Fluorescence spectra were obtained with the excitation wavelength at 280 nm and blue shift in the wavelength of maximum fluorescence (λ_max_) was plotted against the molar ratio of lipid to peptide. (mean ± SE, n = 3).

Fluorescence of Trp is highly sensitive to the local environment; in particular, the emission maximum (λ_max_) of a Trp residue is dependent on its interaction with the membrane bilayer [33, 36]. When PAB-TP was incubated with LUVs composed of DOPA, DOPC, and cholesterol at the molar ratio of 10:60:30, an extensive blue shift in the Trp emission spectra of PAB-TP was observed, while no significant shift was observed upon incubation with the vesicles without PA (Fig 6A). A progressive shift of λ_max_ to shorter wavelength was observed in a dose-dependent manner of the PA-containing vesicles, while no significant shift was seen even with the highest concentration of the vesicles without PA (Fig 6B). Together with the results obtained by the inhibition analysis of SPVB assay (Fig 4A) and the amino acid replacement analysis (Fig 5A), these results indicate that the interaction with the hydrophobic interior of the lipid bilayer is responsible for the stable association of PAB-TP to the membrane. We next reevaluated the interaction between the soluble form of PAB-TP and PA-containing vesicles by measuring the shift in the Trp emission spectra. As shown in Fig 7A, the shift of λ_max_ was highly dependent on the contents of PA in the membrane, which was consistent with the results obtained with the SPVB assay (Fig 2A). Analysis of the phospholipid specificity of PAB-TP in the fluorescence shift assay demonstrated that the peptide showed a weak cross-reaction with PS, ceramide-1-phosphate (C1P), PG, and GM3, but a significant cross-reactivity with PI(4,5)P_2_ was observed (Fig 7B). Quantitative analysis showed that PAB-TP cross-reacted with PI(5)P, PI(4)P and PI(4,5)P_2_ to the same extent as with PA, suggesting that the phosphomonoester head group of phospholipids is required for the high affinity interaction with PAB-TP (Fig 7C). It is noteworthy that PAB-TP exhibited differential reactivity to the phosphomonoester-containing phospholipids, i.e. PA and C1P, implying that the structural or physicochemical properties of C1P might affect the interaction with PAB-TP [37].

In summary, we have established a novel tetravalent peptide PAB-TP that can react with as low as 1 mol% of PA and distinguish the ionization properties and the microenvironment of PA molecules in the membrane. The key features that confer the high affinity binding to PA are the involvement of the Trp residue and the couplings of adjacent Arg and His residues in the binding as well as their multivalent interactions with PA molecules dispersed in the membrane. The Trp residue at position 4 (Fig 1A) was shown to be involved in the interaction with PA by the amino acid replacement analysis (Fig 5A). Although the contribution of a single Arg or His residue is relatively small (Fig 5A), the couplings of adjacent Arg and His residues at positions from 5 to 7 are required for the effective binding (Fig 5B). It is noteworthy here that, Kooijman et al. demonstrated that the hydrogen-bond formation between the phosphate of PA and amino acid residues such as lysine and arginine in proteins increased the charge of PA, thereby stabilizing the protein-lipid interaction [38]. Involvement of His residues in the interaction with the phosphate of phospholipids has been observed with various enzymes such as phospholipase A2 [39] and phospholipase D [40]; a recent study on the crystal structure of *E*. *coli* phosphatidylglycerol-phosphatase B (ecPgpB), a membrane-integrated type II PA phosphatase (PAP2) that catalyzes the dephosphorylation of PA, showed that two His and two Arg residues are involved in the putative phosphate-binding site [41]. The mutations in these residues abolish the catalytic activity of ecPgpB, and these residues are conserved among members of the PAP2 family. With these observations, it is plausible to speculate that a combination of electrostatic and hydrogen bond interactions mediated by the adjacent His and Arg residues with the phosphomonoester head group of PA provide the basis of the high-affinity interaction of PAB-TP with PA as originally proposed for PA-binding proteins by Kooijman [38].

PAB-TP may provide a useful tool to observe the intracellular and transbilayer distribution of PA by using a freeze-fracture replica labeling immunoelectron microscopy [42]. However, further structural analyses to increase the specificity for PA and labeling of the peptide without changing the reactivity of the peptide are required to explore the molecular motion and function of PA in living cell membrane

## Supporting Information

S1 Fig Kinetic analysis of binding of PAB-TP to PA-containing vesicles by QCM-D measurements. LUV layers composed of DOPA/DOPC/biotin-DOPE/cholesterol (10:58:2:30) or DOPC/biotin-DOPE/cholesterol (68:2:30) was incubated with increasing concentrations of PAB-TP ((i) 7.8, (ii) 16, (iii) 63, (iv) 125, (v) 250, (vi) 500, (vii) 1000 nM) for 5 min. After incubation with each concentration of PAB-TP, TBS was introduced to induce the release of PAB-TP for 5 min. Representative result at 35 MHz harmonic was shown.
(TIF)
