# Supplementary figures and images for: Development of a Novel Tetravalent Synthetic Peptide That Binds to Phosphatidic Acid
Source: PLoS One. 2015 Jul 6;10(7):e0131668. doi: 10.1371/journal.pone.0131668 (PMC4493020; doi:10.1371/journal.pone.0131668)

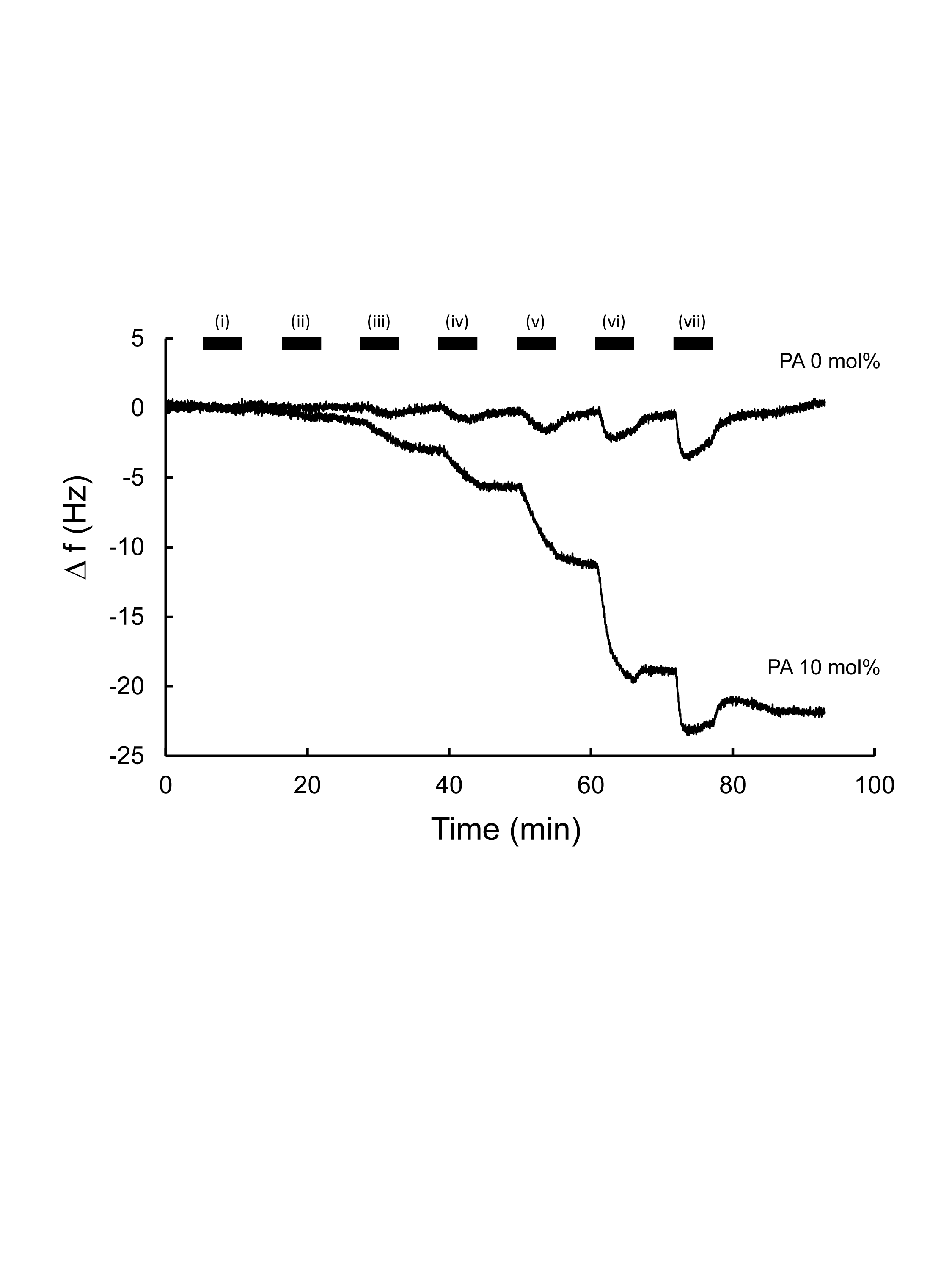

Supplement: S1 Fig — LUV layers composed of DOPA/DOPC/biotin-DOPE/cholesterol (10:58:2:30) or DOPC/biotin-DOPE/cholesterol (68:2:30) was incubated with increasing concentrations of PAB-TP ((i) 7.8, (ii) 16, (iii) 63, (iv) 125, (v) 250, (vi) 500, (vii) 1000 nM) for 5 min. After incubation with each concentration of PAB-TP, TBS was introduced to induce the release of PAB-TP for 5 min. Representative result at 35 MHz harmonic was shown. (TIF) [file pone.0131668.s001.tif]
